# Intestinal Permeability and Cellular Antioxidant Activity of Phenolic Compounds from Mango (*Mangifera indica* cv. Ataulfo) Peels

**DOI:** 10.3390/ijms19020514

**Published:** 2018-02-08

**Authors:** Ramón Pacheco-Ordaz, Marilena Antunes-Ricardo, Janet A. Gutiérrez-Uribe, Gustavo A. González-Aguilar

**Affiliations:** 1 Centro de Investigación en Alimentación y Desarrollo, A.C., Carretera a la Victoria Km 0.6, La Victoria, Hermosillo 83000, Sonora, Mexico; ramon.pacheco@estudiantes.ciad.mx; 2 Tecnologico de Monterrey, Centro de Biotecnologia-FEMSA., Av. Eugenio Garza Sada 2501 Sur, Monterrey C.P. 64849, Nuevo León, Mexico; marilena.antunes@itesm.mx; 3 Tecnologico de Monterrey, Department of Bioengineering and Science, Campus Puebla, Av. Atlixcáyotl 2301, Puebla C.P. 72453, Puebla, Mexico

**Keywords:** cellular antioxidant activity, Caco-2 monolayer, gallic acid, mangiferin, mango by-products, intestinal permeability

## Abstract

Mango (*Mangifera indica* cv. Ataulfo) peel contains bound phenolics that may be released by alkaline or acid hydrolysis and may be converted into less complex molecules. Free phenolics from mango cv. Ataulfo peel were obtained using a methanolic extraction, and their cellular antioxidant activity (CAA) and permeability were compared to those obtained for bound phenolics released by alkaline or acid hydrolysis. Gallic acid was found as a simple phenolic acid after alkaline hydrolysis along with mangiferin isomers and quercetin as aglycone and glycosides. Only gallic acid, ethyl gallate, mangiferin, and quercetin were identified in the acid fraction. The acid and alkaline fractions showed the highest CAA (60.5% and 51.5%) when tested at 125 µg/mL. The value of the apparent permeability coefficient (Papp) across the Caco-2/HT-29 monolayer of gallic acid from the alkaline fraction was higher (2.61 × 10^−6^ cm/s) than in the other fractions and similar to that obtained when tested pure (2.48 × 10^−6^ cm/s). In conclusion, mango peels contain bound phenolic compounds that, after their release, have permeability similar to pure compounds and exert an important CAA. This finding can be applied in the development of nutraceuticals using this important by-product from the mango processing industry.

## 1. Introduction

The consumption of tropical fruits has increased in the last decade due to the concern that consumers have to live a healthier life style [1,2]. Mango (*Mangifera indica* L.) is one of the most consumed fruits worldwide for its sensory properties and for being considered a good source of bioactive compounds such as ascorbic acid, carotenoids, tocopherols, and phenolic compounds (PC). PC are involved in the aroma and flavor of plant foods, and are responsible for plant defense against infections [3]. In addition, these compounds are associated with different health benefits such as antioxidant, anti-inflammatory, anti-microbial, and anti-proliferative effects [4,5].

Mango cv. Ataulfo is a widely consumed Mexican variety, and has the highest PC content and antioxidant capacity compared with other varieties [6]. However, only the pulp of the fruit is consumed, and it is consumed either fresh or processed into purees, juices, jams, or canned slices. Mango processing generates a large amount of by-products such as peels and kernels, which account for 35–65% of the total fruit [7]. However, the peels and kernels are discarded into the environment as waste, making them a source of pollution.

Lately, the use of food by-products as a source of phytochemicals has increased. Quercetin, kaempferol, and mangiferin from the extracts of the leaves and bark extracts are extensively used as dietary supplements due to their health benefits. Unlike pulp, which contains mainly small molecules like phenolic acids [8], more complex molecules, such as quercetin, catechin, mangiferin, gallotannins, gallic acid, and other glycosylated compounds have been identified in mango peel, which indicates that it could be a source of functional ingredients [9,10]. Several authors have studied the potential benefits of mango waste.

In nature, most PCs exist linked to other complex structures such as proteins and fiber. Therefore, pre-treatments are necessary before conventional extraction to obtain the complete phenolic profile of the plant. Generally, alkali treatment can break the ester bonds that linked the PC to the cell wall components and release the majority of bound phenolics. In contrast, acid hydrolysis breaks glycoside bounds and therefore releases the aglycones [11].

The beneficial effects of the PC depend on their absorption throughout the gastrointestinal tract [12,13]. Several in vivo and in vitro studies describe the absorption of PC. Among them, the human colon adenocarcinoma (Caco-2) monolayer model has been widely used in recent years for the screening of the permeability of different drugs [14].

The major challenge in the development of nutraceuticals is their bioavailability, since, in nature, these compounds interact within the food matrix in their conjugated forms [15]. In addition, the plant matrix or the other components have been reported to alter the pharmacokinetics of PC and therefore their bioactivity as antioxidant agents. In this study, the PCs’ profile and the biological activities of three (03) different fractions of mango peel extract were analyzed. The aim of this study was to evaluate the intestinal permeability of free and bound PCs present in mango cv. Ataulfo fractions after an alkaline and acid hydrolysis using a Caco-2 monolayer model, as well as, to evaluate the contribution of these free and bound PCs in the cellular antioxidant activity of each fraction in order to determine the potential of this by-product as a source of nutraceuticals.

## 2. Results

### 2.1. Identification and Quantification of PCs in Mango cv. Ataulfo Peel Extracts

**Figure 1:**
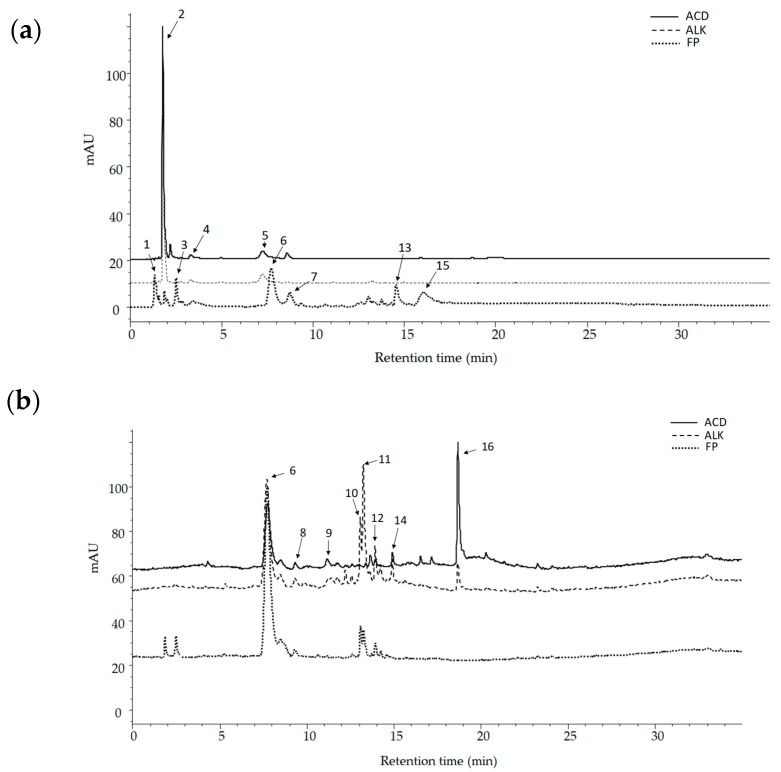
Base peak HPLC chromatograms at (**a**) 280 nm and (**b**) 365 nm with the identification of phenolic compounds detected in free phenolic (FP), alkaline (ALK), and acid (ACD) fractions obtained from mango cv. Ataulfo peel.

Of the PC found in the alkaline (ALK) and acid (ACD) extracts, gallic acid (peak 2) was the major component (Figure 1a). Mangiferin (peak 6) was among the most abundant PC in the free phenolics extract (FP), and a significant increase in quercetin (peak 16) was observed in the extract following acid hydrolysis (ACD) (Figure 1b). Additionally, other compounds were identified in the different extracts based on their UV maxima and accurate mass (Table 1). In FP, compounds with complex structure such as gallotannins, xanthones, glycosylated flavonoids, and other phenolic acid derivatives were identified based on preliminary reports [16,17]. The gallotannins were identified as galloyl glycoside (peak 1) with an [M–H]^−^ ion at *m*/*z* 331, hyemaloside A (peak 7) with an [M–H]^−^ ion at *m*/*z* of 728, methyl digallate ester (peak 13) with an [M–H]^−^ ion at *m*/*z* of 335, and hexagalloyl glucoside (peak 15) with a [M–H]^−^ ion at *m*/*z* of 1091. The quercetin hexoside I and II (peak 10 and peak 12) [M–H]^−^ ion at *m*/*z* 463 were found in the FP extract.

Instead of the free gallic acid (peak 2) found in ALK fraction, three gallic acid derivatives were identified in FP: galloyl quinic acid (peak 3), digallic acid (peak 5), and ethyl gallate (peak 8) with a [M–H]^−^ ion at *m*/*z* of 343, 321, and 197, respectively. Three mangiferin isomers appeared at 365 nm (peaks 6, 11, and 14) with a [M–H]^−^ ion at *m*/*z* of 421 each one.

In addition to the gallic acid release, other phenolic acids, such as caffeoyl hydroxycitric acid (peak 4) and coumaric acid (peak 9), were extracted after alkaline hydrolysis. The alglycone of quercetin (peak 16) was also identified in this fraction and showed a [M–H]^−^ ion at *m*/*z* of 301. In contrast, acid hydrolysis released fewer compounds; only gallic acid, ethyl gallate, mangiferin, and quercetin were identified in ACD fraction.

### 2.2. Cytotoxicity and Cellular Antioxidant Activity (CAA) of Mango cv. Ataulfo Peel Extracts

Among the three tested extracts (FP, ALK, and ACD), FP was the most cytotoxic followed by ALK (Table 2). Caco-2 and HT-29 cells in combination were more sensitive to the three mango peel extracts.

**Figure 2:**
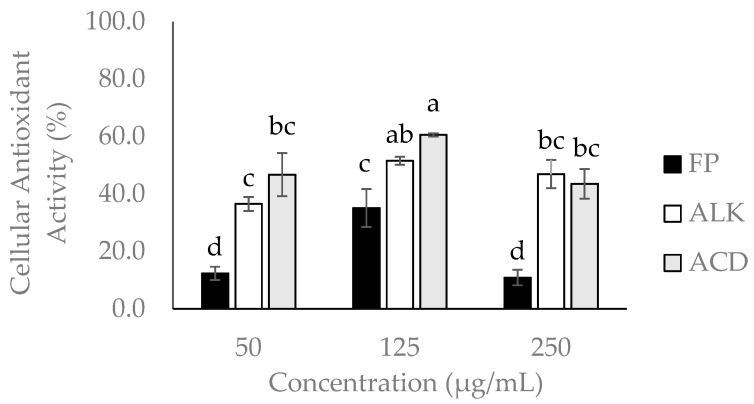
Cellular antioxidant activity (CAA) of the free phenolic (FP), alkaline (ALK), and acid (ACD) fractions obtained from mango cv. Ataulfo peel. Caco-2 cells were treated with 50, 125, and 250 µg/mL of each fraction for 20 min. Value are presented as mean ± standard deviation; a, b, c, d different letters above the bars indicate significant differences in the PC content between mango peel fractions (*p* < 0.05).

The ACD and ALK fractions showed the highest antioxidant potential (60.57 ± 0.55% and 51.56 ± 1.39%) at 125 µg/mL. Regarding to the FP fraction, it showed a value of IC_50_ about 2.0-fold lower than the maximum concentration assayed for CAA (250 µg/mL), and therefore Caco-2 cells viability was strongly affected, and in consequence it showed lower antioxidant activity (10.90%) than the obtained when tested at 125 µg/mL (35.17%) (Figure 2).

### 2.3. Intestinal Permeability Experiment

**Figure 3:**
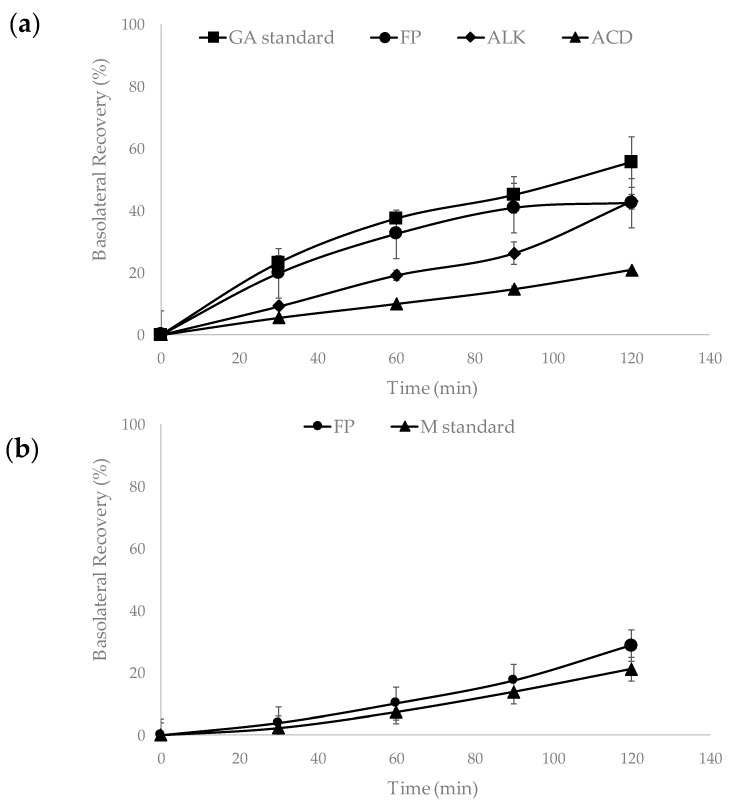
Basolateral recovery (%) of phenolic compounds from free phenolic (FP), alkaline (ALK) and acid (ACD) fractions obtained from mango cv. Ataulfo peel, gallic acid (GA), and mangiferin (M) standards, after permeability experiments in a Caco-2/HT-29 cell monolayer. (**a**) Basolateral recovery of gallic acid and (**b**) basolateral recovery of mangiferin.

The basolateral side of the intestinal permeability experiment represents the basal surface of the membrane that mediates the transport of nutrients from cell to surrounding fluids that lead to the circulatory system. Gallic acid and mangiferin were detected in this side of the Caco-2/HT-29 (75:25%) monolayer after 30 min of incubation with the extract (Figure 3a,b). Basolateral recovery of gallic acid ranged between (20.8 ± 0.63%)–(55.6 ± 8.14%) for all samples (Figure 3a). In contrast, mangiferin was only detected in the FP fraction, with a recovery of 28.84 ± 0.28% (Figure 3b). The FP and ALK fractions presented similar recovery values for gallic acid 42.39 ± 1.76% and 43.02 ± 2.38%, respectively. The gallic acid standard showed the highest recovery percentage (Figure 3a). The mass balance obtained for gallic acid in ALK, ACD fractions, with gallic acid standard, was between 94–98%, and in the case of FP it was of 55 ± 2.5%. On the other hand, mangiferin standard exhibited an average mass balance of 88%.

**Figure 4:**
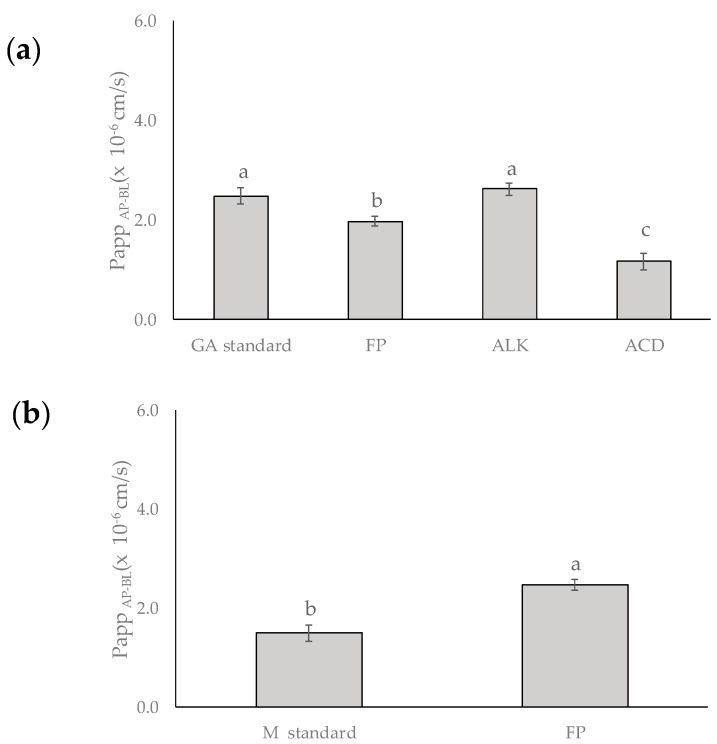
Apparent permeability (Papp) of (**a**) gallic acid (GA) and (**b**) mangiferin (M) present in free phenolic (FP), alkaline (ALK), and acid (ACD) fractions obtained from mango cv. Ataulfo peel, and their respective standards from apical to basolateral compartment (AP-BL).

The values of the apparent permeability coefficient (Papp) of gallic acid in the FP, ALK, and ACD using the Caco-2/HT-29 co-culture were 1.97, 2.61, and 1.16 × 10^−6^ cm/s, respectively (Figure 4a). The gallic acid standard showed a higher (Papp of 2.48 × 10^−6^ cm/s) value compared with the FP and ACD but was similar to the obtained for gallic acid found in ALK mango peel extract. On the other hand, the Papp value for the mangiferin standard was 1.49 × 10^−6^ cm/s, whereas the value of the FP fraction was 2.47 × 10^−6^ cm/s (Figure 4b).

## 3. Discussion

### 3.1. Identification of PCs of Mango cv. Ataulfo Peel Extract

Since phytochemicals are mainly found bound to proteins, fiber, or other complex structures in nature; alkaline and acid hydrolysis were carried out to fully characterize the mango cv. Ataulfo peel extract. The most abundant family of compounds identified in mango extract fractions were gallotaninns, mangiferin isomers, and flavonoids. Gallic acid derivatives such as galloyl glucose, methyl digallate ester, and methyl gallate were also detected. These results agree with those reported by López-Cobo et al. [10], which indicated that gallotanins and other gallic acid conjugates were found in high concentrations in the peels of mango of the Keitt, Osteen, and Sensation cultivars. Similarly, Dorta et al. [20] demonstrated the presence of gallotannins, ethyl gallate, methyl gallate, gallic acid, galloyl glucose, and theogallin, as well as the xanthone mangiferin, in mango peel. Additionally, López-Cobo et al. [10] identified quercetin glycosides such as isoquercitrin, quercetin-3-*O*-galactoside, and quercetin pentoside, which is consistent with the results of this study.

Gómez-Caravaca et al. [18] analyzed four parts of mango Keitt, and the peel of the tissue with the highest content of PC. The majority of the compounds identified in the peel were phenolic acids such as gallic, syringic, protocatechuic, and ferulic acid ellagic acid, as well as ellagic acid derivatives. However, in this study, only gallic and caffeic acids conjugates were found. Differences between the profiles of PC among mango varieties and cultivars depend on multiple factors including the type of soil, weather, temperature, hydric stress, and post-harvest damage, all of which affect the biosynthetic pathway of PC in plants [6,15].

### 3.2. Cytotoxicity of Mango Peel Extract

The FP fraction was the most cytotoxic to Caco-2, HT-29 cell lines, and the mixture of the two. This may be explained by the abundance of gallotanins in this fraction [23]. Urueña et al. [25] demonstrated that a gallotannin-rich fraction obtained from *Caesalpinia spinosa* reduced the proliferation of breast cancer through the activation of apoptosis pathway, resulting in the activation of caspases. Likewise, gallotanins may regulate the production of reactive oxygen species (ROS) by altering the redox balance in the cell, which activates the intrinsic mitochondrial apoptosis pathway [26]. Mangiferin can induced apoptosis by activating caspase-8, caspase-9, and caspase-3 and pro-apoptotic protein Bid. Also, reductions in the activation of nuclear factor-kappaB (NF-κB) can caused a decrease in matrix metalloproteinase-7 and -9, and inhibit the β-catenin pathway [27]. Additionally, the presence of some flavonoids and mangiferin could be causing the synergistic antiproliferative effects observed in this sample [28].

### 3.3. Cellular Antioxidant Activity (CAA)

The ACD fraction was the more effective in the reduction of intracellular reactive oxygen species (ROS) production, which was monitored based on the Dichloro-dihydro-fluorescein diacetate (DFFH-DA) fluorescence assay. The major compounds detected in ACD were quercetin and gallic acid, and both compounds have demonstrated a high antioxidant activity. Differences in CAA between FP and ACD can be explained by the abundance of glycosylated PC in the FP fraction. In previous studies, quercetin showed the highest antioxidant activity in comparison with its glycosylated form due to the availability of hydroxyl groups to participate in antioxidant reactions [24,25,26]. On the other hand, an hormesis effect was observed when the FP and ACD fraction were tested at the concentration of 250 µg/mL. Hormesis is defined as a biphasic dose-dependent response in which a compound in low doses results in an antioxidant effect, while it became pro-oxidant at high concentration levels [28]. PC can exert a pro-oxidant effect by formatting labile aroxyl radical and reacting with oxygen producing superoxide anion (O_2_^−^) [29]. This effect has been observed in quercetin and in others PCs [30,31,32].

Abbasi et al. [33] assessed the cellular antioxidant activity of the mango pulp and peel from nine cultivars in the liver hepatocellular cell line (HepG2). Mango peel extracts showed the highest antioxidant activity (2986.5 ± 380 µmol QE/100 g FW.) The cultivar Xiao Tainang showed the highest CAA with an IC_50_ of 130 µg/mL, whereas in our study, ACD fraction of mango cv. Ataulfo peel at the concentration of 125 µg/mL showed 60% of CAA. PCs can exert their antioxidant activity in two ways; they can act at the cell membrane and break peroxyl radical chain reactions at the cell surface, or they can enter the cell and react intracellularly with ROS. Polar compounds can interact with the membrane surfaces by hydrogen bonding and protecting cell membranes from external and internal oxidative stress [34]. On the other hand, hydrophobic compounds can be embedded more easily in the membrane and influence the fluidity and disrupt oxidative chain reactions. The difference in CAA observed between FP, ALK, and ACD is related with the permeability of the phenolic profile of each fraction. This could be an explanation of why the ACD and ALK fractions in general showed more antioxidant potential than the FP fraction.

### 3.4. Instetinal Permeability Assay

Caco-2/HT-29 monolayer has been used to evaluate the permeability of new drugs and phytochemicals due to its similarity to the human intestinal epithelium, and the combination of the two cell lines decreased the expression of P-gp transporters in Caco-2 allowing the permeability of xenobiotics. The apparent permeability (Papp) values of tannin corilagin and gallic acid were higher when tested pure than in an extract, because PC can act antagonistically and obstruct the absorption of other compounds [35,36,37]. On the contrary, in our study ALK exhibited similar Papp to the gallic acid standard. This could be due to the fact that other specific compounds in the extract can improve the absorption of gallic acid as reported by Xie et al. [38].

In the present study, free gallic acid was not detected in the FP but it was found in the basolateral side 30 min into the monolayer permeability assay. An explanation could be that the FP is rich in gallotannins and these compounds could enter into the cell by the action of the specific transporters and become degraded intracellularly to gallic [39,40]. It has already been reported that gallotannins such as penta-galloylglucose can be degraded to tri- and tetra-galloylglucose by esterases from Caco-2 cells while they are being transported across cell monolayer [41].

## 4. Materials and Methods

### 4.1. Material and Methods

Mango cv. Ataulfo fruits (commercial ripeness stage) that were free from external defects were purchased from a local market (Hermosillo, Sonora, México) and transported to the laboratory. Mangos were washed with tap water, sanitized, and peeled. Fruit peels was freeze-dried (LABCONCO, Kansas City, MO, USA), subsequently ground, and stored at −35 °C until analysis.

### 4.2. Chemicals and Reagents

Gallic acid and mangiferin standards, 2,2′-azobis-(2-methylpropionamidine) dihydrochloride (AAPH), Lucifer yellow (LY), 2′,7′-dichlorofluorescin diacetate (DCFH-DA), water, formic acid, and acetonitrile HPLC grade were obtained from Sigma-Aldrich, Inc. (St. Louis, MO, USA). Dubelcco’s Modified Eagle Medium (DMEM-F12) and McCoy’s medium were obtained from Thermo Fisher Scientific (Waltham, MA, USA). Fetal bovine serum (FBS), Hank’s Balanced Salt Solution (HBSS), Phosphate Saline Buffer pH 7.4 (PBS), Trypsin-EDTA 0.25%, penicillin (10,000 Unit/mL), and streptomycin (10,000 μg/mL) were acquired from GIBCO (Grand Island, NY, USA). CellTiter 96^®^ AQueous One Solution Cell Proliferation Assay was obtained from Promega Corporation (Madison, WI, USA).

### 4.3. Phenolics Extraction Procedure

**Figure 5:**
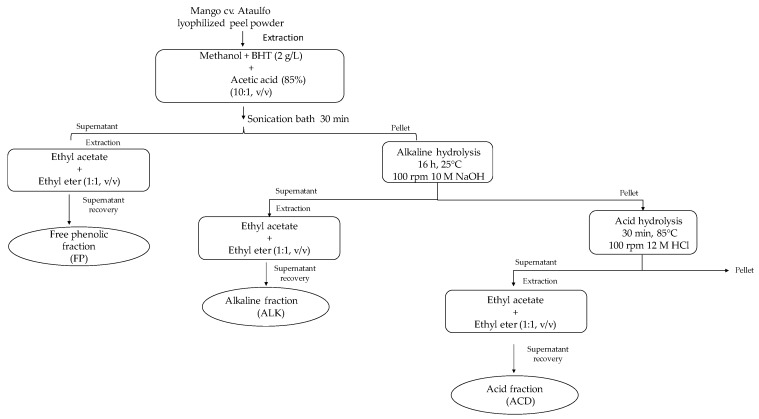
Schematic description of the extraction procedure to obtain free phenolic (FP), alkaline (ALK), and acid (ACD) fractions from mango cv. Ataulfo peel.

The extraction of phenolic compounds was carried out following the method described by Mattila and Kumpulainen [42], with modifications (Figure 5). Dried mango cv. Ataulfo peel (500 mg) was weighted and homogenized with 7 mL of a mixture of ethanol and 10% acetic acid using a magnetic stirrer. Afterwards, peel sample was ultrasonicated for 30 min (Bransonic Ultrasonic Co., Danbury, CT, USA) and centrifuged at 10,000 rpm for 10 min, then the supernatant was recovered. This supernatant was considered the free phenolic rich-fraction (FP) and 1 mL was taken directly for HPLC analysis.

A mixture of 12 mL distilled water and 5 mL of 10 M NaOH were added to the remaining mango peel (pellet). The sample was immediately flushed with nitrogen, then sealed and stirred at 100 rpm and room temperature for 16 h. The solution was then adjusted to pH 2 with 12 N HCl and extracted three times with 15 mL of a mixture of diethyl ether and ethyl acetate (1:1; *v*/*v*). The supernatant of each extraction was recovered, pooled, and evaporated to dryness with a rotary evaporator (R-3000 Buchi, Flawil, Switzerland) and finally resuspended in 1.5 mL of ethanol. This sample was defined as alkaline fraction (ALK) and aliquot was taken for the HPLC analysis. The residual sample was subsequently hydrolyzed in acid by adding 2.5 mL of 10 M HCl and incubating the mixture in a water bath (Thermo Fisher Scientific, Waltham, MA, USA) at 85 °C for 30 min. The sample was left to cool down at room temperature and the pH was adjusted to 2. Same extraction described above with diethyl ether and ethyl acetate was used for the hydrolyzed product. This sample identified as acid fraction (ACD), and an aliquot was taken for the HPLC analysis.

### 4.4. High Performance Liquid Chromatography (HPLC) Analysis

PC of mango cv. Ataulfo peel were identified by HPLC coupled to DAD-UV detector (1200 Series, Agilent Technologies, Santa Clara, CA, USA) using the method reported by Acosta-Estrada [43] with some modifications. Separation were carried out using a Zorbax SB-Aq, 3.0 mm × 100 mm (3.5 μm) reverse phase column at a temperature of 25 °C with a flow rate 0.6 mL/min. The mobile phase used was (A) water pH 2 acidified with formic acid and (B) acetonitrile 100%. The gradient started with 5% of B and increased to 30% within the first 15 min, changed to 60% at 20 min, then to 80% 5 min after, and at 30 min B increased to 100%. Chromatograms were obtained at 280 nm and 365 nm after the injection of 5 μL of sample and integrated by HP-Agilent Software (Chemstation for LC Copyright Agilent Technologies, Santa Clara, CA, USA).

The identification of the PC was performed on a liquid chromatography coupled with time-of-flight mass spectrometry (LC/MS-TOF) (Agilent Technologies, Santa Clara, CA, USA) and equipped with a quaternary pump system with a vacuum degasser, a thermostated column compartment with an electrospray ionization source (ESI). The same conditions describe above were used. Mass spectra were acquired in negative mode over a range from 150 to 1500 *m/z.*

### 4.5. Cell Culture

Human colorectal adenocarcinoma cells (Caco-2 and HT-29) were obtained from American Type Culture Collection (ATCC) (Manassas, VA). Caco-2 cells were grown in DMEM-F12 supplemented with 5% fetal bovine serum, whereas HT-29 cells were grown in McCoy’s medium supplemented with 10% fetal bovine serum. Cells were incubated at 37 °C in a humidified atmosphere containing 5% CO_2_.

### 4.6. Cytotoxicity Assay

The effect of mango cv. Ataulfo peel free phenolics extract (FP) and the corresponding alkaline and acid hydrolysates (ALK and ACD) on cell viability was measured with CellTiter 96 Aqueous One Solution Cell Proliferation Assay (Promega, Madison, WI). Caco-2 and HT-29 were seeded individually and combined (Caco-2/HT-29; 75:25%) in a 96-well plate in a solution of 100 µL at 5 × 10^5^ cells/mL. After 24 h, the extracts were added at final concentrations ranging between 50 and 500 µg/mL. After 48 h of incubation, 20 µL of CellTiter 96 was added to each well so the cell viability could be determined by measuring the absorbance at 490 nm in a microplate reader (Synergy HT, Bio-Tek, Winooski, VT, USA). The IC_50_ values (concentration to inhibit by 50%) for each sample were determined.

### 4.7. Cellular Antioxidant Activity (CAA) Assay

The cellular antioxidant activity (CAA) assay was performed as reported for López-Barrios et al. [44]. A day before the experiment, Caco-2 cells were cultured in a black-walled, clear-bottom 96-well microplate (Costar, Corning Inc., Corning, NY, USA) at a density of 5 × 10^5^/mL. After 24 h, medium was removed and cells were washed with 100 µL of phosphate buffered saline (PBS). Afterwards, cells were treated with 100 µL of the FP, ALK, and ACD fractions of mango peel extract at different concentrations (50, 125, and 250 µg/mL) containing DCFH-DA (60 μM), and the cells were incubated at 37 °C for 20 min. Following incubation, the treatment solutions were removed and the cells were washed twice with PBS. Finally, 100 µL of 500 μM AAPH solution was added to each well, except for blank and negative control wells. The microplate was placed in the microplate reader (Synergy HT, Bio-Tek, Winooski, VT, USA). Fluorescence emitted at 538 nm with excitation at 485 nm was measured every 2 min for 90 min at 37 °C. The CAA values of mango peel extract at each concentration were calculated using the following Equation (1).

(1) CAA unit=1−(∫SA/∫CA)

where ∫SA is the integrated area under the sample fluorescence versus time curve and ∫CA is the integrated area from the control curve.

### 4.8. Caco-2/HT-29 Monolayer Permeability Assay

A Caco-2 and HT-29 co-culture (ratio 75:25%, respectively) was seeded at a density of 1 × 10^5^ cells/insert in trans-well inserts (polycarbonate membrane, 12 mm i.d., 1.12 cm^2^ growth area, 0.4 µm pore size (Corning, NY, USA)) and placed in 6 well plates with 1.5 mL of medium at the apical side (AP) and 2.5 mL at the basolateral side (BL). Cells were allowed to grow and differentiate for 21 days to form a monolayer, and the culture medium was replaced three times per week. The permeability experiment was carried out according to Antunes-Ricardo et al. [45]. The medium was removed and cell monolayers were washed with Hank’s balanced salt solution (HBSS). FP, ALK, and ACD, as well as gallic acid and mangiferin standards, were inoculated in the apical side of the differentiated Caco-2/H-T29 monolayers. The experiment was carried out for 120 min, and the samples were withdrawn from apical side and along with the basolateral samples taken at 30, 60, 90, and 120 min they were analyzed by HPLC-DAD-UV.

The apical side was filled with 0.4 mL of LY solution (100 µM) and the basolateral side was filled with HBSS. After 2 h, 100 µL of basolateral and apical media were transferred to a 96-well plate and the fluorescence of each sample was measured at 530 nm (emission) and 485 nm (excitation) using a microplate reader. Cell monolayers’ integrity was monitored by lucifer yellow (LY) permeation [46] using the apparent permeability coefficient (Papp) value, which is calculated with the following Equation (2):

Papp = (*dQ*/*dt*) × (*V*/A*C_0_) (2)

where *dQ*/*dt* is the change in drug concentration in the receiver solution (µM/s), *V* represents the volume of the solution in the receiving compartment (mL), *A* denotes the membrane surface area (cm^2^), and *C*_0_ is the initial concentration in the donor compartment (µM). Data from membranes displaying an LY apparent permeability coefficient (Papp) >1 × 10^−6^ cm/s were excluded. The LY concentration was calculated on the basis of a standard curve in the concentration range of 1.56 to 100 µM.

## 5. Statistical Analysis

All experiments were performed at least three times, and results are expressed as the mean ± standard deviation. Statistical analyses were performed using the statistical software JMP 13.0 (SAS Institute Inc., Cary, NC, USA). Data were analyzed by ANOVA followed by Tukey’s HSD test with a significance level of *p* < 0.05.

## 6. Conclusions

Mangiferin, gallic acid, and quercetin were the main compounds identified in mango peel extracts. Gallic acid released after alkaline hydrolysis had similar permeability to that obtained with a pure standard. The ALK fraction showed better permeability properties than the other fraction and a greater antioxidant potential, due to its high content of PCs. The dose tested and the structure of the PC play an important role in their permeability and therefore in their antioxidant activity. The use of three different extracts allowed us to obtain a better characterization of PC profile and compare their biological activity. Although the extraction after alkaline hydrolysis will need to be evaluate to determine if its suitable for industrial set up, due the large amount of solvents and recovery yield, this finding provides novel and valuable information that can be applied in the development of nutraceuticals from mango peels. Based on our results, compounds such as tannins and glycosides were not absorbed, which indicate that they can reach the colon and possibly modify the bacteria populations present in this part of the gastro-intestinal tract. In this sense, further studies are needed in order to assess the prebiotic potential of mango cv. Ataulfo peel.

## Figures and Tables

**Table 1:** Phenolic compounds found in free phenolics (FP), alkaline (ALK), and acid (ACD) hydrolysis extracts from mango *Mangifera indica* cv. Ataulfo peel.

Peak Number	UV Max	Accurate Mass	*m*/*z* (M–H)^−^	Molecular Formula	Tentative Identification	Concentration (µg/mg)			Reference
						FP	ALK	ACD	
1	272	332.07	331	C_13_H_16_O_10_	Galloyl glycoside ^a^	0.81	*NQ*	*NQ*	[16]
2	270	170.02	169	C_7_H_6_O_5_	Gallic acid ^a^	*NQ*	271.46	184.56	[18]
3	282	344.07	343	C_14_H_16_O_10_	Galloyl quinic acid ^a^	3.24	*NQ*	*NQ*	[19]
4	280	370.05	369	C_15_H_14_O_11_	Caffeoyl hydroxycitric acid ^a^	*NQ*	10.71	6.82	[19]
5	275	322.03	321	C_14_H_10_O_9_	Digallic acid ^a^	*NQ*	40.29	23.22	[18]
6	238, 256, 318, 365	422.08	421	C_19_H_18_O_11_	Mangiferin ^b^	36.02	*NQ*	7.11	[18]
7	280	728.12	727	C_33_H_28_O_19_	Hyemaloside A ^a^	3.19	*NQ*	*NQ*	[17]
8	271	198.05	197	C_9_H_10_O_5_	Ethyl gallate ^a^	*NQ*	1.69	10.07	[20]
9	227, 309	164.05	163	C_9_H_8_O_3_	Coumaric acid ^a^	*NQ*	2.23	1.39	[21]
10	257, 352	464.09	463	C_21_H_20_O_12_	Quercetin hexoside I ^c^	0.54	0.56	*NQ*	[10]
11	241, 259, 317, 367	422.08	421	C_19_H_18_O_11_	Mangiferin isomer I ^a^	2.33	5.38	*NQ*	[22]
12	256, 351	464.09	463	C_21_H_20_O_12_	Quercetin hexoside II ^c^	0.23	0.37	*NQ*	[16]
13	280	336.05	335	C_15_H_12_O_9_	Methyl digallate ester ^a^	4.22	*NQ*	*NQ*	[16]
14	236, 261, 318, 369	422.08	421	C_19_H_18_O_11_	Mangiferin isomer II ^a^	*NQ*	0.71	0.07	[22]
15	279	1092.13	1091	C_48_H_36_O_30_	Hexagalloyl glucose ^a^	7.28	*NQ*	*NQ*	[23]
16	254, 370	302.04	301	C_15_H_10_O_7_	Quercetin ^c^	*NQ*	0.27	1.44	[24]
					Sum	57.86	333.67	234.68	

*NQ*: The compound was detected under the limits of quantification; ^a^ Quantified as µg of gallic acid equivalents; ^b^ Quantified as µg of mangiferin equivalents; ^c^ Quantified as µg of quercetin equivalents.

**Table 2:** Antiproliferative activity of Mango cv. Ataulfo peel extracts fractions, against Caco-2, HT-29 cell lines, and in combination 75:25%. Values were expressed as the half maximal inhibitory concentration (IC_50_) in µg/mL.

Mango Extract	Cell Line											
	Caco-2				HT-29				Caco-2/HT-29 (75:25)			
Free Phenolic	135.03	±	23.39	Cb	190.50	±	11.45	Ca	135.84	±	13.34	Bb
Alkaline H	246.46	±	4.83	Ba	227.86	±	3.32	Bb	157.38	±	6.64	Bc
Acid H.	327.91	±	12.89	Aa	261.05	±	5.03	Ab	235.29	±	3.84	Ac

A, B, C Capital letters indicate difference between the extracts. a, b, c Small letters indicate differences between cell lines (*p* < 0.05).

## Abbreviations

CAA	Cellular antioxidant activity
PC	Phenolic compounds
FP	Free phenolic fraction
ALK	Alkaline fraction
ACD	Acid fraction
GA	Gallic acid
M	Mangiferin
ROS	Reactive oxygen species
DFFH-DA	Dichloro-dihydro-fluorescein diacetate
AAPH	2,2′-azobis-(2-methylpropionamidine) dihydrochloride
Papp	Apparent permeability coefficient
Papp_AP-BL_	Apparent permeability coefficients from apical to basolateral direction
